# ﻿Independent origins of Spiranthes×kapnosperia (Orchidaceae) and their nomenclatural implications

**DOI:** 10.3897/phytokeys.226.100062

**Published:** 2023-05-19

**Authors:** Matthew C. Pace

**Affiliations:** 1 New York Botanical Garden, 2900 Southern Blvd., Bronx, New York, 10348, USA New York Botanical Garden New York United States of America

**Keywords:** hybrid speciation, Interior Lowlands, nomenclatural priority, species complex, *Spiranthescernua*, *Spiranthesochroleuca*

## Abstract

*Spiranthes* Rich. (Orchidaceae) is a commonly encountered but systematically and nomenclaturally challenging component of the North American orchid flora. Here, the evolutionary history and hybrid origin of the recently described *S.sheviakii* Hough and Young are critically examined. The available molecular data unambiguously support a hybrid origin of *S.cernua* (L.) Rich. × *S.ochroleuca* (Rydb.) Rydb. for *S.sheviakii*, the same parentage as the priority name S.×kapnosperia M.C. Pace. As hybrid formulas can have only one correct name, *S.sheviakii* is a synonym of S.×kapnosperia. It is likely that S.×kapnosperia evolved independently at least twice in at least two widely disjunct locations.

Species complexes continue to present some of the most impenetrable systematic challenges for evolutionary biology and conservation biology, and the challenges associated with their study are amplified when species within a complex hybridize (e.g., Arnold 2001; Fu et al. 2020), with challenging implications for nomenclature. Although Orchidaceae have been long seen as a model family for pre-zygotic barriers to hybridization, primarily due to documented or inferred pollinator specificity (Ackerman et al. 2023), a growing body of literature makes clear that reproductive barriers are often porous, and that hybridization plays an important role in the speciation of many orchid genera (e.g., *Dactylorhiza* Neck. ex Nevski (e.g., Pillon et al. 2007), *Epidendrum* (e.g., Pinheiro et al. 2010), *Ophrys* L. (e.g., Soliva and Widmer 2003), *Orchis* Tourn. ex L. (e.g., Jacquemyn et al. 2012), *Platanthera* Rich. (e.g., Wettewa et al. 2020), and *Tolumnia* Raf. (e.g., Ackerman and Galarza-Pérez 1991)), making for ‘fuzzy’ species boundaries.

*Spiranthes* is one such orchid genus where renewed systematic attention has supported many previous hypotheses of hybridization (e.g., Dueck et al. 2014), in addition to the discovery of new hybrid taxa (e.g., Pace and Cameron 2017). Of the 44 currently accepted *Spiranthes* species (including nothospecies), 10 have molecular evidence to support a hybrid origin (Sun 1996; Arft and Ranker 1998; Szalanski et a. 2001, Dueck et al. 2014; Pace 2015, 2021; Pace and Cameron 2017, 2019; Surveswaran et al. 2018; Pace et al. 2019). These hybrid species do not only occur within complexes of closely related species (e.g., S.×stellata P.M.Br., Dueck and K.M.Cameron), but between clades of species complexes that are often distantly related (e.g., *S.diluvialis* Sheviak). The *S.cernua* (L.) Rich. species complex has traditionally been regarded as systematically “intractable” (Sheviak 1982, 1991; Sheviak and Brown 2002), primarily due to the frequency of hybridization and cryptic speciation (Pace and Cameron 2017) and the variability of all taxa involved. Within the *S.cernua* species complex, the identity of *S.ochroleuca* (Rydb.) Rydb. has contributed significantly to systematic and nomenclatural challenges. For example, primarily due to the nature of the *Gyrostachysochroleuca* Rydb. holotype (*Mrs Long s.n.*, drawing, NY barcode 9463, Fig. 1), and morphological similarities to other members of the complex, *S.ochroleuca* has either been treated as a synonym or variety of *S.cernua* for much of the last 90 years (e.g., Gleason and Cronquist 1963). It was only after the detailed work of Sheviak and Catling (1980), that *S.ochroleuca* was widely accepted as a species fully distinct from *S.cernua* (e.g., Pace and Freudenstein 2018). Despite this distinction, *S.cernua* s.s. and *S.ochroleuca* were still hypothesized to engage in frequent and widespread hybridization and introgression (Sheviak 1982; Sheviak and Brown 2002). Pace and Cameron (2017) presented the first molecular evidence for hybridization between *S.ochroleuca* and *S.cernua* s.s. in the southern Appalachians, describing this hybrid taxon as S.×kapnosperia M.C. Pace.

**Figure 1.**
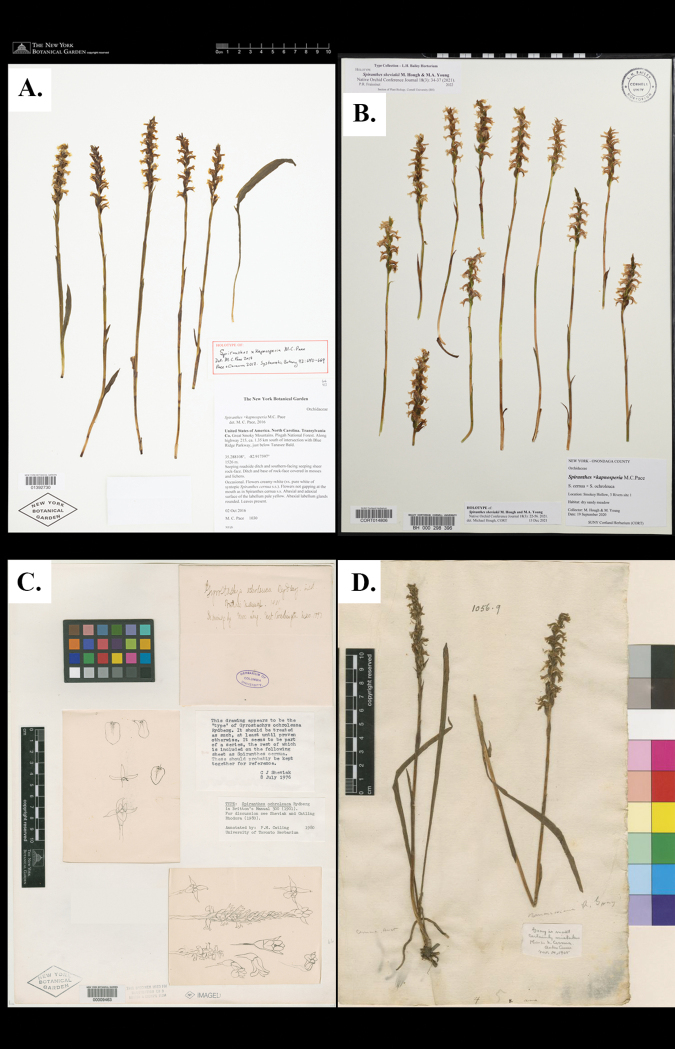
Comparison of type specimens **A** holotype of Spiranthes×kapnosperia (*M.C. Pace 1030*, NY) **B** holotype of *Spiranthessheviakii* (*M. Hough and M.A. Young s.n.*, BH) **C** holotype of *Gyrostachysochroleuca* Rybd. (*Mrs Long s.n.*, NY); this image is a composite of two images to show the front and back of the drawing plate **D** lectotype of *Ophryscernua* L. (*P. Kalm s.n.*, LINN) **A** and **C** courtesy of the C. V. Starr Virtual Herbarium (http://sweetgum.nybg.org/science/vh/) **B** courtesy of the Liberty Hyde Bailey Hortorium, Cornell University **D** courtesy of the Linnean Society of London.

The name *S.sheviakii*Hough and Young (2021) was recently described as a species of hybrid origin distributed from central New York to the greater Ohio River Valley, but Hough and Young (2021) were unusually vague about the parentage of *S.sheviakii*, writing “[this is] apparently the result of hybridization of *S.ochroleuca* with another member of the *S.cernua* species complex” (pg. 47). They included comparisons to *S.cernua*, *S.ochroleuca*, and S.×kapnosperia in the diagnosis and throughout the discussion, noting that *S.sheviakii* is “intermediate in form” between *S.cernua* and *S.ochroleuca* (pg. 37), but did not give a full parentage to their newly proposed species. Curiously, Hough originally identified the type specimens of *S.sheviakii* as “S.×kapnosperia, *S.cernua* × *S.ochroleuca*” (Fig. 1), indicating he was aware of its full parentage, or that he thought these plants were morphologically similar to S.×kapnosperia.

After reviewing the relevant type specimens (Fig. 1) and the publicly available molecular data presented in Pace and Cameron (2017) and Hough and Young (2021), it is clear that both S.×kapnosperia and *S.sheviakii* share a hybrid ancestry of *S.cernua* × *S.ochroleuca*, although the genetic patterns are differently expressed in the resulting regional hybrids. Appalachian S.×kapnosperia displays a discordance between nuclear and chloroplast datasets: the chloroplast data (including *ndhJ*) indicates a maternal parent of *S.ochroleuca*, whereas the nuclear data (*ACO* and nrITS) indicate a paternal parentage of *S.cernua* (Table 2; Pace and Cameron 2017). The *ACO* dataset for Appalachian S.×kapnosperia lacks major nucleotide ambiguities at points of differentiation between the parental species, sharing all of the unique molecular synapomorphies of *S.cernua* vs. *S.ochroleuca*. *Spiranthessheviakii* also displays a discordance between nuclear and chloroplast data, although the discordance is slightly different than in Appalachian S.×kapnosperia. The available *ndhJ* data for *S.sheviakii* clearly indicate a maternal parentage of *S.cernua*, as the samples share all of the same nucleotide patterns as *S.cernua* and are clearly different from *S.ochroleuca* (or any other member of the *S.cernua* species complex). However, *S.sheviakii* displays *ACO* nucleotide ambiguities at all of the exact and unique points of molecular differentiation between *S.cernua* and *S.ochroleuca* (Fig. 2). These ambiguities in the *ACO* dataset indicate a hybrid origin between *S.cernua* and *S.ochroleuca* for *S.sheviakii.* Additionally, the *ACO* nucleotide ambiguity patterns for *S.sheviakii* are distinct from those of regionally sympatric *S.incurva* (Jenn.) M.C. Pace, *S.magnicamporum* Sheviak, or any other member of the *S.cernua* species complex (Fig. 2), indicating these species are not involved in the evolution of *S.sheviakii.* The nucleotide ambiguity and nuclear/chloroplast discordant patterns are consistent across all samples of S.×kapnosperia and *S.sheviakii* included in Pace and Cameron (2017) and Hough and Young (2021). Thus, S.×kapnosperia and *S.sheviakii* share the same ancestral hybrid parentage of *S.cernua* × *S.ochroleuca*, but this parentage is expressed differently within the genomes of the two resulting regionally distinct hybrid populations (Table 2). Nomenclaturally, per The Code (Article H.4.1):

**Figure 2.**
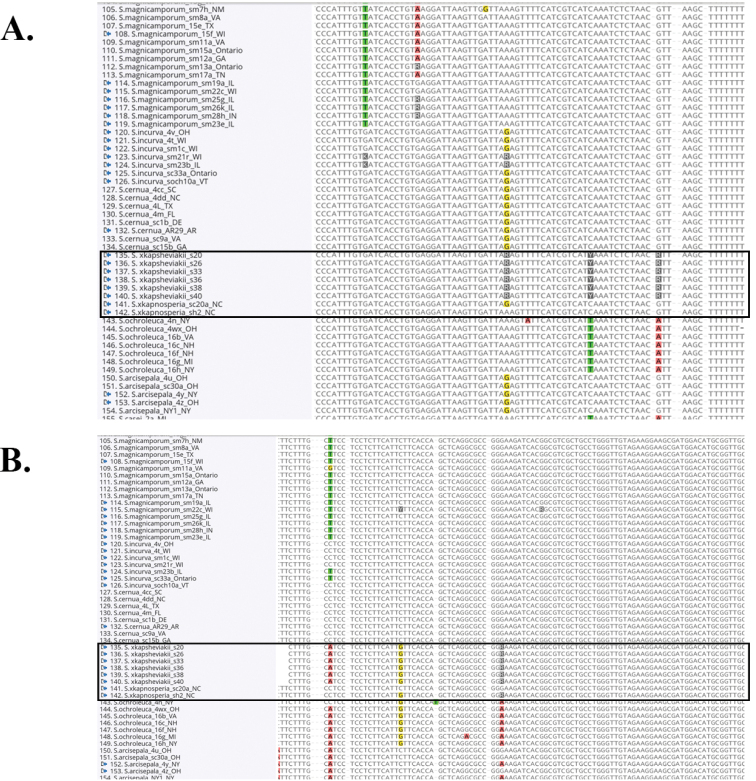
Examples of *ACO* gene sequence concatenations for selected *Spiranthes*. Samples 135–140 labeled “S. ×kapsheviakii” represent *a priori* interior lowland *S.sheviakii* from Hough and Young (2021), all other samples are from Pace and Cameron (2017). Samples 141 & 142 represent Appalachian S.×kapnosperia. Samples of *S.cernua* s.s. are included immediately above the highlighted box and samples of *S.ochroleuca* are included immediately below the highlighted box **A** examples of ambiguities in *a priori S*. *sheviakii* that correspond to nucleotide differences between *S.cernua* and *S.ochroleuca* (e.g., G, R, A) **B** examples of nucleotide states that are shared with *S.ochroleuca* but not *S.cernua* (e.g., left-most highlighted A & G), and additional examples of ambiguous states in *a priori S*. *sheviakii* that correspond to nucleotide differences between *S.cernua* and *S.ochroleuca* (e.g., G, R, and A).

When all the parent taxa can be postulated or are known, a nothotaxon is circumscribed so as to include all individuals recognizably derived from the crossing of representatives of the stated parent taxa (i.e. not only the F1 but subsequent filial generations and also back-crosses and combinations of these). There can thus be only one correct name corresponding to a particular hybrid formula; this is the earliest legitimate name (Art. 6.5) at the appropriate rank (Art. H.5), and other names corresponding to the same hybrid formula are synonyms of it.

Thus, any recognizably intermediate individual or population that results from the hybridization of *S.cernua* and *S.ochroleuca* must be recognized by the priority name S.×kapnosperia, even if different hybridization events between the parental species occurred at different geologic times, in different places, resulting in different genomic expressions, and different morphologies. Based on the available *ndhJ* and *ACO* molecular data of Hough and Young (2021), *S.sheviakii* is unambiguously of hybrid origin between *S.cernua* and *S.ochroleuca*, and is thus synonymous with S.×kapnosperia. It should be noted that species and nothospecies are the same nomenclatural rank, and the use of the multiplication symbol (×) is simply to emphasize the hybrid origin of nothospecies. Because Hough and Young (2021) appear to have been aware of the full hybrid parentage of their newly proposed name when they described *S.sheviakii* (based on the label of the type specimens, *Hough s.n*., Fig. 1), this name is likely superfluous, although it is not illegitimate as they did not include the type of S.×kapnosperia within the circumscription of *S.sheviakii.*

The evolutionary history of S.×kapnosperia in its newly expanded understanding (S.×kapnosperia sensu nov.) is perhaps one of the more unusual within the entire genus, having formed from the same two parental species (at least) two times, in widely disjunct locations, displaying different molecular signals between the parents. Additionally, the parental species likely played different maternal vs. paternal roles in the formation of the regionally disjunct S.×kapnosperia populations (Table 2). The resulting differences in ambiguity patterns (or the lack of ambiguities) are likely due to differences in the hybridization and introgression histories of these regional populations. As Appalachian S.×kapnosperia lacks *ACO* and nrITS ambiguities, it may be the result of chloroplast capture. This is a process through which an initial F1 hybridization event between paternal *S.cernua* and maternal *S.ochroleuca* is then followed by several backcrossing events with *S.cernua* as the pollen (paternal) parent, until the entire nuclear genome is only represented by *S.cernua*, but the chloroplast genome retains the original chloroplast contribution of *S.ochroleuca*. By contrast, the ambiguities present in the *ACO* locus of Interior Lowland S.×kapnosperia (previously referred to as *S.sheviakii*) indicate it likely resulted from an initial F1 hybridization without extensive (or only limited) backcrossing. Elsewhere in *Spiranthes*, Arft and Ranker (1998) hypothesized that at least two separate hybridization events between *S.magnicamporum* and *S.romanzoffiana* Cham. led to the formation of *S.diluvialis*, with subsequent localized dispersal in Utah and Colorado. However, the examined molecular signals from all sampled populations were the same (at the time of their study *S.diluvialis* was known from Colorado, Montana, Nevada, Utah, and Wyoming, but their study focused on samples from Colorado and Utah; Arft and Ranker 1998). Additional molecular phylogenetic study has not found major molecular differentiation between different populations of *S.diluvialis* (Dueck et al. 2014; Pace 2015).

Spiranthes×kapnosperia was originally known to occur diffusely over a small region of the greater Smoky Mountain region and southern Blue Ridge Mountains, in the southern Appalachian Mountains of North Carolina, South Carolina, and Tennessee (Pace and Cameron 2017). The expanded understanding of S.×kapnosperia sensu nov. discussed here extends the known distribution of this nothospecies throughout the distributional contact zone between *S.cernua* and *S.ochroleuca* along the northern limit of *S.cernua* in the area of the Interior Lowlands, Ohio River Valley, and southern Great Lakes Basin, an area that was not heavily sampled in the molecular work of Pace and Cameron (2017). Ecologically, populations in the southern Appalachians occur in more mesic sites vs. less mesic habitat of the Interior Lowlands populations; habitat variability is not uncommon across the genus. Morphologically, both disjunct populations are readily identifiable as intermediate hybrids of *S.cernua* × *S.ochroleuca*. However, they display slightly different morphological affinities to their parents, with southern Appalachian S.×kapnosperia being more similar to *S.ochroleuca*, and Interior Lowland S.×kapnosperia being more similar to *S.cernua*. The flowers of southern Appalachian S.×kapnosperia are generally slightly ascending (as is common in *S.ochroleuca*). The flowers of Interior Lowlands S.×kapnosperia are generally very similar in overall size and appearance to *S.cernua* s.s., commonly with a nod to the flowers, but sharing the yellowish labellum coloration and rounded abaxial labellum glands with *S.ochroleuca* (Table 1).

**Table 1.** Comparison of *Spiranthescernua*, S.×kapnosperia, and *S.ochroleuca*.

Taxon	Distribution	Flower color	Flower position	Labellum color (Adaxial / Abaxial)	Abaxial gland shape	Lateral sepal position
*S.cernua*	Maritime Canada s. to n. FL, west to central PA, the interior lowland plateaus, and e. Texas	White	Perpendicular to stem to strongly nodding	White to very pale yellow / white	Conical, reduced	Sepal base held in-line with the profile of the flower, sepals ascending
S.×kapnosperia	Southern Appalachian Mountains of NC, SC, and TN; southern Great Lakes Basin from central NY to IL, s. to Ohio River Valley	White to ivory	Nodding to slightly ascending	Pale yellow / pale yellow	Rounded	Sepal base held just below the profile of the flower, sepals ascending
*S.ochroleuca*	Maritime Canada s. to w. NC, west through the Southern Great Lakes Basin, disjunct in s. IL, IN, central KY, and TN	Ivory to ochroleucous	Slightly to strongly ascending	Deep yellow /deep yellow	Rounded (sometimes reddish)	Sepal base held below the profile of the flower, held low against the profile of the flower, downwardly falcate to ascending

**Table 2.** Inferred genetic contributions for S.×kapnosperia sensu nov. Chloroplast regions include *matK*, *ndhJ*, *trnL-F*, *trnS-M*, and *ycf1* 3’ (Pace and Cameron 2017). The only chloroplast region sampled in Hough and Young (2021) for a priori *S.sheviakii* is *ndhJ*.

Hybrid taxa	ACO (nuclear)	nrITS	Chloroplast
S.×kapnosperia (Appalachian S.×kapnosperia)	*S.cernua* s.s.	*S.cernua* s.s.	*S.ochroleuca*
*S.sheviakii* (Interior Lowland S.×kapnosperia)	*S.cernua* s.s. + *S.ochroleuca*	[not sampled]	*S.cernua* s.s.

Within the *S.cernua* species complex, molecular data have supported hybridization as a strong driver of speciation, with four of the seven non-hybrid species within the complex involved in the evolution of six species of hybrid origin or nothospecies (Table 3, Pace and Cameron 2017). *Spiranthescernua* is the most frequently involved species, giving rise to the evolution of four hybrid taxa, and is typically the inferred maternal parent. The frequent involvement of *S.cernua* in the evolution of hybrid taxa may be due to its broad geographic distribution, stretching from Maritime Canada south to northern Florida and west through the mid- and southern-Appalachian Mountains to Texas. The repeated evolution of hybrid taxa such as S.×kapnosperia, in addition to the cryptic morphological nature of the species within the complex, has contributed to the systematic and nomenclatural challenges commonly associated with the genus. The repeated evolution of S.×kapnosperia and complicated hybridization history of the wider *S.cernua* species complex also highlight the need for slow and careful study when deciding to name and describe new taxa within the genus (Ames 1921), with particular attention given to nomenclatural rules and priority. Although they do not provide any molecular data, Hough and Young (2021), using terminology from Sheviak (1982), also place emphasis on the potential distinction of “low prairie race” and “southern prairie complex” populations currently contained within the circumscription of *S.cernua* s.s. Moving forward, researchers should keep in mind the priority names for taxa that involve hybridization between *S.cernua* and other members of the *S.cernua* species complex (Table 3). Additionally, not all individuals or populations of hybrid ancestry should be named, as genomic data increasingly shows complex hybridization and introgression patterns make for cryptically complex genetic ancestries and species relationships in groups with porous reproductive barriers (e.g., Evans et al. 2023).

**Table 3.** Ancestry of known hybrid taxa derived from members of the *S.cernua* species complex, as supported by combined molecular and morphological evidence.

Hybrid taxa	Inferred paternal species	Inferred maternal species	Literature source
*S.bightensis*	*S.odorata*	*S.cernua*	Pace (2021)
*S.casei*	*S.lacera* (var. lacera)	*S.ochroleuca*	Pace (2015); Pace unpublished data
*S.diluvialis*	*S.magnicamporum*	*S.romanzoffiana*	Arft and Ranker (1998)
*S.incurva*	*S.magnicamporum*	*S.cernua*	Pace and Cameron (2017)
S.×kapnosperia sensu nov.	*S.cernua* (southern Appalachian populations) or *S.ochroleuca* (Interior Lowland populations)	*S.cernua* (Interior Lowland populations) or *S.ochroleuca* (southern Appalachian populations)	Pace and Cameron (2017); Hough and Young (2021)
*S.niklasii*	*S.cernua*	*S.ovalis*	Pace and Cameron (2017)

A few additional notes related to Hough and Young (2021) are discussed here: 1) Hough and Young (2021) discuss the “holotype for *S.incurva*.” As detailed in Pace and Cameron (2017), the name *S.incurva* is a nomenclatural combination based on the basionym *Ibidiumincurvum* Jenn.:

Since Jennings selected a suite of specimens, “Aug. 24–26, 1905”, housed at CM as “the type specimens”, and not a specific specimen, collection number, or sheet, the specimen designated by Catling as the holotype, via an annotation label, is more properly designated as the lectotype. All other specimens collected on Aug. 26, 1905 must then be isolectotypes, and all other specimens collected within “the type specimens” collection range designated as syntypes.

The lectotype of *I.incurvum* is the *Jennings s.n.* specimen collected on 26 August 1905, from Fog Whistle (CM). However, this specimen is not discussed or examined in Hough and Young (2021), which only discusses the remaining syntypes. 2) Hough and Young (2021) note “at least within the range of this study, we have not observed *S.incurva* growing in xeric sites. The typical habitats appear to be mostly moist to wet and mediacid to calcareous.” It should be noted that *S.incurva* is found in a wide variety of habitats, from hot, dry, sandy lake beach dunes, old fields, and roadside embankments, to standing in shallow water of fens and lake beach dune swales (Pace and Cameron 2017). This is inclusive of locations within the study range of Hough and Young (2021). 3) Hough and Young (2021) make much of apparent ambiguities in the nuclear *ACO* data for their accessions of *S.arcisepala* M.C. Pace, claiming that Pace and Cameron (2017) misinterpreted their data. After comparing the GenBank data of Hough and Young (2021) to the raw sequence data of Pace and Cameron (2017) (Fig. 3), there was a single instance where Pace and Cameron (2017) missed an ambiguity that is present in the data of Hough and Young (2021). However, the overwhelming majority of supposed *S.arcisepala* ambiguities present in Hough and Young (2021) are simply not present in our data, which are unambiguously a single nucleotide. Furthermore, I found an additional ambiguity in the *ACO* data of Pace and Cameron (2017) that is not present in the *ACO* data of Hough and Young (2021), and this point of ambiguity does not correspond to a point of molecular differentiation between any other member of the *S.cernua* species complex (Fig. 3). Based on this reexamination and comparison, I reassert that *S.arcisepala* is not of hybrid origin, although it may be an autopolyploid. Genomic examination of hybridization across the genus is obviously needed, and is currently underway.

**Figure 3.**
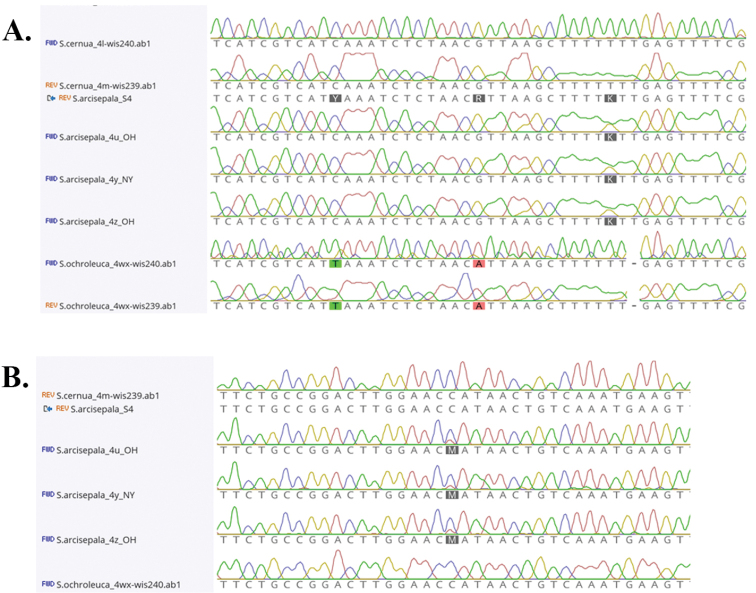
Examples of *ACO* gene nucleotide ambiguities for selected *Spiranthes*. The sample “S.arcisepala_S4” is from Hough and Young (2021), all other samples are from Pace and Cameron (2017)**A** examples of ambiguities present in Hough and Young (2021), but not present in Pace and Cameron (2017) (Y and R), and an ambiguity present in Hough and Young (2021) but overlooked in Pace and Cameron (2017) (K) **B** example of ambiguity present in Pace and Cameron (2017) (M), but not present in Hough and Young (2021).

## ﻿Nomenclature

### ﻿ Spiranthes × kapnosperia

Taxon classificationPlantaeAsparagalesOrchidaceae

M.C. Pace [S. cernua × S. ochroleuca], Syst. Bot. 42: 659. 2017.

B741205C-EB43-5D9D-B8EB-DBC9ED8091A4

= Spiranthessheviakii M. Hough and M.A. Young. Native Orchid Conf. J. 18.3: 35. 2021. Type: U.S.A. New York: Onondaga County, town of Lysander, Three Rivers WNA, 19 Sep 2020, *M. Hough and M.A. Young s.n.* (holotype: BH! [BH 000298396]; isotype: CORT! (×2)).

#### Type.

U.S.A. North Carolina: Transylvania County, Great Smoky Mountains, Pisgah National Forest, ca 7.5 km NW of Balsam Grove, north side of 215, below a steep seeping cliff, growing in moss and lichen hummocks, 2 Oct 2016, *M.C. Pace 1030* (holotype: NY! [01392730]; isotype: NCU! [NCU00332163], US!).

## Supplementary Material

XML Treatment for Spiranthes × kapnosperia
